# Spatial Spillover Effects of Air Pollution on the Health Expenditure of Rural Residents: Based on Spatial Durbin Model

**DOI:** 10.3390/ijerph18137058

**Published:** 2021-07-01

**Authors:** Bo Sun, Bo Wang

**Affiliations:** 1 School of Economics and Management, Huzhou University, Huzhou 313000, China; 02761@zjhu.edu.cn; 2 Business School, Guangxi University, Nanning 530004, China

**Keywords:** PM2.5, health expenditure, spatial Durbin model, spatial spillover effect, environmental protection

## Abstract

Background: Air pollution is one source of harm to the health of residents, and the impact of air pollution on health expenditure has become a hot topic worldwide. However, few studies aim at the spatial spillover effects of air pollution on the health expenditure of rural residents (HE-RR), including the impact on the health expenditure in neighboring areas. Objective: Based on the existing research, this paper further introduces the spatial dimension and uses the Spatial Durbin model to discuss the impact of environmental pollution on the health expenditure of rural residents (HE-RR). Methods: Based on provincial panel data during 2002–2015 in China, the Spatial Durbin model was used to investigate the spatial spillover effect of the average annual concentration of PM2.5 (AAC-PM2.5) on the health expenditure of rural residents (HE-RR). Results: There was a significant positive correlation between AAC-PM2.5 and health expenditure of rural residents (HE-RR) in neighboring areas at a significant level of 5% (COEF: 2.546, Z: 2.340), that is, AAC-PM2.5 has a spatial spillover effect on PC-HE-RR in neighboring areas, and the spatial spillover effect is greater than the direct effect. The migration and diffusion of PM2.5 pollution will affect the air quality of neighboring areas, leading to the health risk not only from the local PM2.5 pollution but also the nearby PM2.5 pollution. Conclusion: The results show a significant positive relationship between air pollution and HE-RR in neighboring areas, and the spatial spillover effect is greater than the direct effect.

## 1. Introduction

Remarkable economic progress has been accompanied by serious environmental problems and consequent high health costs in China. According to the National Cancer Center (2019), lung cancer and upper digestive system tumors associated with air pollution are still the primary malignant tumors in China. In addition, with the growing concern of Chinese people about health problems and the increasingly severe problem of population aging in China, the health expenditure of Chinese residents also shows a rapid growth trend.

Firstly, air pollution has a direct and significant negative impact on the health of residents. For example, particulate matter in the air and toxic elements related to inhalable particulate matter can affect heart rate variability, blood pressure, vascular tension, blood coagulation, and atherosclerosis (Miao et al., 2010; Shan et al., 2016; Muhammad, 2018) [1,2,3], leading to respiratory symptoms, cardiovascular problems, lung cancer, lung dysfunction, and asthma (Joel, 2007; Xu et al., 2017; Zhen, 2018) [4,5,6], even causing severe case mortality (Michael B, 2018; Pablo F, 2017; Antonis A, 2018) [7,8,9]. In addition, He (2018) found that fine particles in the air are the main contributors to the burden of disease and disability-adjusted life years [10]. Jing (2017) thinks that besides daily air pollution, the continuous impact of high-level air pollution on the mortality rate for several days is also huge [11]. Some scholars have put forward some countermeasures and suggestions for this problem. For example, the improvement of medical and health services can reduce the risk of air pollution damage to health (Sun et al., 2017 [12]; Qu et al., 2015 [13]). Furthermore, individual choice of residents regarding medical insurance and environmental protection behavior can also effectively reduce the health damage caused by environmental pollution (Chen et al., 2017) [14].

Secondly, there is a significant positive correlation between air pollution and the health expenditure of residents. The deterioration of air quality leads to the increase of health expenditure of residents (Mao et al., 2016 [15]; Ye, 2013 [16]; Li, 2018 [17]). This influence is caused by environmental degradation affecting health levels and stimulating demand for medical and health services (Ouyang et al., 2017) [18]. Zhang et al., (2020) thought that the damage of air pollution to health could further inhibit economic growth [19]. Xie (2014) [20], Chen et al., (2010) [21], Xie (2016) [22] measured the major health losses caused by PM2.5 or PM10 pollution into economic losses. Yang et al., (2017) found that reducing industrial COD and SO_2_ emissions during the study period is beneficial to reduce the health expenditure of residents [23]. Muhammad (2019) thinks that environmental pollution has a significant positive impact on health expenditure, but environmental pollution has no significant effect on health expenditure in the short term [24]. Despina (2018) studied 59 countries worldwide and found that reducing agricultural ammonia emissions by 50% can prevent 200,000 people from dying every year, with billions of dollars in economic benefits [25]. Yazdi (2017) used the Autoregressive distributed lag (ARDL) method to find that carbon dioxide emission in MENA countries had statistically significant positive effects on health expenditure from 1995 to 2014 [26]. Furthermore, through the analysis of different groups, Zhao (2020) [27] found that air pollution significantly increased the medical expenses of middle-aged and elderly people. The increase of the dependency ratio of the elderly and the decrease of the dependency ratio of the teenagers will lead to the rise of the health expenses of residents (Ye et al., 2014) [28].

Thirdly, the impact of air pollution on health expenditure also shows certain regional differences in China. Wang et al., (2019) [29] found through panel model research that the emission of sulfur dioxide and nitrogen oxides in air pollution data in southern China will inhibit the growth of public health expenditure, while the emission of smoke (dust) will promote the growth of public health expenditure, while the emission of sulfur dioxide and smoke (dust) in northern China will promote the growth of public health expenditure. Zhang (2019) [30] found that environmental pollution significantly increased per capita medical insurance expenditure based on three dimensions: waste gas, solid waste, and wastewater, and the impact of environmental pollution on per capita medical insurance expenditure in the eastern region was significantly greater than that in the central and western regions.

To sum up, air pollution indirectly affects the health expenditure of residents by directly harming health, and this influence also shows significant regional differences in China. Furthermore, few researchers such as Li (2019) [31] found that the health risks in a region have some relationship with PM2.5 pollutions in neighboring areas, which means that air pollution has certain spatial transmission and diffusion. Therefore, based on the existing research, this paper further introduces the spatial dimension, and discusses the impact of environmental pollution on HE-RR by using the spatial Durbin model. This paper is divided into four parts. The first part is the introduction, which expounds on the research background and existing research results; The second part is data and variables, explaining the problem of data source and variable selection; The third part is the empirical results and analysis. The fourth part is empirical conclusions and policy recommendations.

## 2. Materials and Methods

It is necessary to take individual spatial distribution into the investigation dimension and conduct empirical research from the perspective of spatial spillover in the study of the relationship between air pollution and the health expenditure of residents.

### 2.1. Spatial Durbin Model

Before understanding the possible spatial correlation and specific characteristics of the relationship between the proportion of the health expenditure in consumption expenditure of rural residents and AAC-PM2.5, we need to test the spatial autocorrelation of variables to examine whether spatial correlation exists or not. In the existing academic research, there are mainly two methods for spatial autocorrelation tests: Moran index and Geary index. Among them, the Moran index is the earliest and most widely used spatial autocorrelation test method, which is often used to test the similarity or difference between sample regions or independent. Therefore, this paper also uses the Moran index to test the spatial correlation of variables. Moran’s index ranges from −1 to 1. If Moran’s I > 0, there is a positive spatial correlation. The larger the value is, the stronger the positive spatial correlation is; If Moran’s I = 0, then there is no spatial autocorrelation. If Moran’s I < 0, it means that the spatial correlation is negative. The larger the value is the more significant the spatial repulsion.

According to the existing research, socio-economic variables such as the economic situation of the individual, the emission of other pollutants, the accessibility of medical resources, the dependency ratio of the elderly and the young are all important factors affecting the per capita health care expenditure of residents. This paper innovatively introduces the environmental pollution factors and draws on the empirical model of Li (2019) and other scholars. The following model (Formula (1)) is constructed for empirical analysis.

(1) $$y_{i,t}=\alpha +\beta_{1}ln\rho_{i,t}+\beta_{2}Wln\rho_{i,t}+X^{\prime }\gamma +WX^{\prime }\omega +\varepsilon_{i,t}$$

In Formula (1), i is the provincial cross-sectional unit, t is the year, y is the proportion of the health expenditure in consumption expenditure of rural residents, $\;\rho_{i,t}$ is the concentration of PM2.5, W is the spatial weight matrix reflecting the spatial adjacency, $\beta_{1}$ reflects the influence of AAC-PM2.5 in local area on the variable *y*, $\beta_{2}$ reflects the influence of AAC-PM2.5 in the adjacent area on the variable *y*, and $X^{\prime }$ is the matrix of related factors affecting the proportion of the health expenditure in consumption expenditure of rural residents; γ is the coefficient matrix of the control variable; ω is the coefficient matrix of the spatial delay term of the control variable, $\varepsilon_{i,t}$ is the random perturbation.

### 2.2. Variable Selection and Data Source

#### 2.2.1. Dependent Variable

The proportion of the health expenditure in consumption expenditure of rural residents. Combined with previous studies such as Xie et al., (2018) [32] and the availability of data, this paper chooses the proportion of the health expenditure in consumption expenditure of rural residents as the dependent variable; namely, the proportion of per capita cash payment in per capita annual consumption expenditure when rural residents receive various health services, which reflects the burden of health expenses of rural residents.

#### 2.2.2. Core Independent Variables 

Environmental pollution variables, which mainly represent by AAC-PM2.5, also include industrial wastewater discharge per unit area (10,000 tons) and industrial waste gas discharge per unit area (100 million standard cubic meters). In previous studies, the measurement indicators of air pollution in various places were generally annual average PM10 concentration, smoke and dust emissions, sulfur dioxide emissions, carbon dioxide emissions, and per capita nitrogen oxide emissions. In this paper, PM2.5 annual average concentration is selected as the core independent variable, which is rare in previous studies. The annual average concentration of PM2.5 is processed by first-order lag and logarithm considering the lag effect of air pollution on health.

**Figure 1:**
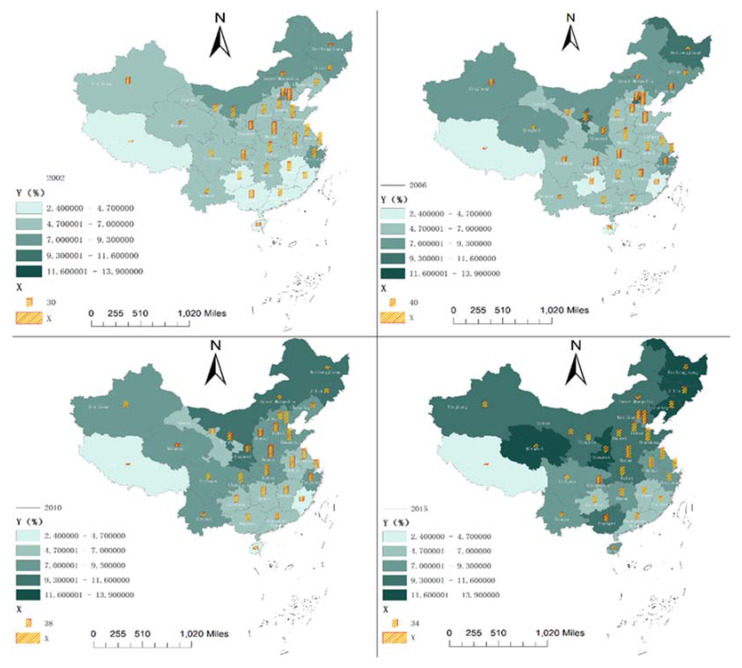
Spatial distribution of AAC-PM2.5 and HE-RR in China in 2002, 2006, 2010, and 2015.

Figure 1 shows the spatial distribution of HE-RR and AAC-PM2.5 in China in 2002, 2006, 2010, and 2015, respectively. The color of each province represents the level of HE-RR in the province, and the darker the color, the greater the level of HE-RR.; The length of the stacking map in each province represents AAC-PM2.5. The longer the stacking map, the higher AAC-PM2.5.

AAC-PM2.5 increased year by year from 2002 to 2015, and there were also differences among provinces, among which, the lowest concentration of PM2.5 in Tibet. In 2002, PM2.5 high concentration provinces were concentrated in Heilongjiang, Jilin, and Inner Mongolia in Northeast China; By 2006, AAC-PM2.5 of Heilongjiang Province was further deepened, and the PM2.5 pollution in Xinjiang, Qinghai, Ningxia, and Shaanxi was further strengthened; In 2010, AAC-PM2.5 of each region continued to increase. By 2015, Qinghai, Heilongjiang, Jilin, and Shaanxi had become the most polluted area in China.

In terms of the proportion of the health expenditure of rural residents to consumption expenditure, the difference between different years is not apparent, but in the same year, the proportion of the health expenditure of rural residents in Shandong, Henan, Jiangsu, Tianjin, and other places is significantly higher than that of other provinces in China. The proportion of the health expenditure of rural residents to the consumption expenditure shows a trend of decreasing from coastal areas to inland areas.

In addition, the previous studies show that the economic situation of the individual family, the accessibility of medical resources, population structure, market-oriented degree are also important factors affecting the medical and health expenditure of rural residents. This paper selects the Engel coefficient of rural residents, namely the proportion of food expenditure in total consumption expenditure in the household, as the control variable reflecting the economic situation of the residents. Compared with the average income in some previous studies, the Engel coefficient of rural residents can reflect their income and expenditure and objectively reflect their actual economic situation. Other control variables include the number of health technicians per 10,000 population, the number of beds in medical and health institutions per 10,000 people, and the total dependency ratio (the sum of the elderly care ratio and the juvenile dependency ratio).

## 3. Results

### 3.1. Descriptive Statistics of Variables

This study selected 31 provinces of China as the research object, intercepted the provincial panel data of China from 2002 to 2015, a total of 434 samples, to study the impact of air pollution (PM2.5 annual average concentration) on the per capita health expenditure of rural residents in China. The data of PM2.5 comes from the Atmospheric Composition Analysis Group of Dalhousie University. Like the data released by Columbia University, this data is the one-year mean value of PM2.5 comprehensively estimated by the data of NASA satellite and ground monitoring station.

The data of industrial wastewater discharge per unit area and industrial waste gas discharge per unit area are derived from China Statistical Yearbook and China Environmental Statistics Yearbook. The independent variables and control variables are respectively derived from the China Health and family planning statistical yearbook, China Statistical Yearbook, and China Environmental Statistics Yearbook. Descriptive statistical analysis of each variable is shown in Table 1.

### 3.2. Panel Data Regression

This paper uses the data of 31 provinces in China from 2002 to 2015, t = 14, *n* = 31, which belongs to the short panel data. Through the Hausman test, this paper uses the individual fixed effect model to estimate the parameters of the panel data. Next, according to the collected and estimated data, panel data regression is carried out, and the regression results are shown in Table 2.

From the regression results of the panel data model in Table 2, AAC-PM2.5 in each region has a positive effect on the proportion of the health expenditure in consumption expenditure of local rural residents (at the 5% significance level). However, the Engel coefficient of rural residents has a negative impact on the proportion of the health expenditure in consumption expenditure of local rural residents (at a significant level of 1%). This conclusion is consistent with the difficulty and high cost of seeing a doctor for a few rural residents in China. The accessibility of medical resources (the number of health technicians per 10,000 people) has a positive effect on the proportion of the health expenditure in consumption expenditure of local rural residents, and the increase of health personnel is conducive to rural residents to receive medical and health services. In the current empirical study, the impact of industrial waste gas and wastewater emissions on health is negligible. The influence of the number of beds per 10,000 people and the total dependency ratio on the proportion of health expenditure to consumption expenditure of local rural residents is not significant (Sickbed, *p*-value = 0.124; Dr, *p*-value = 0.747). 

Furthermore, PM2.5 has a significant positive relationship with per capita health expenditure (Coef. = 0.862, *p*-value = 0.018). Therefore, reducing PM2.5 is conducive to maintaining public health and reducing per capita health expenditure of residents. The increase of family income provides fundamental support for rural residents to receive health care services, and increasing the number of professional medical staff is more conducive to residents to receive health care services. In order to improve the rapid growth of domestic health expenditure and realize the rational use of medical resources, the relevant departments can improve the current situation of environmental pollution, improve the medical insurance system, and vigorously cultivate professional medical personnel, so as to promote healthy development of national medical and health expenditure.

### 3.3. Spatial Econometric Analysis

#### 3.3.1. Spatial Autocorrelation Test

In this paper, the inverse distance square weight matrix is used to test the proportion of the health expenditure of rural residents in consumption expenditure and the spatial dependence of PM2.5 pollution. After the inverse distance square weight matrix is obtained, the final spatial weight matrix W is obtained after row normalization. Table 3 shows the Moran index values of the proportion of the health expenditure of rural residents in consumption expenditure and PM2.5 annual average concentration in various regions of China from 2002 to 2015, as well as other spatial autocorrelation test indicators. It can be seen from Table 3 that the Moran index of the proportion of the health expenditure of rural residents in consumption expenditure fluctuated around 0.2 from 2002 to 2015. Most of the years are significant at 1% significance level, and all the years are significant at 10% significance level. The results show that a significant positive spatial correlation in the proportion of the health expenditure of rural residents to consumption expenditure in China, and this spatial correlation has shown a trend of decreasing volatility since 2010. In the spatial autocorrelation test of annual average PM2.5 concentration in various regions of China, the results show that the Moran index of annual average PM2.5 concentration in all regions of China fluctuated around 0.2 from 2002 to 2015, and the Moran index was generally larger than the Moran index of the proportion of the health expenditure in consumption expenditure of rural residents, and was significant at the significance level of 1%, It also shows that there is a spatial correlation between the annual average PM2.5 concentrations in various regions of China. From the trend point of view, this correlation is fluctuating growth and continues to increase.

The results of spatial autocorrelation test show that there is spatial autocorrelation between Moran index and PM2.5 concentration in rural areas. Therefore, it is reasonable and necessary to use spatial econometric model to analyze variables.

#### 3.3.2. Regression Results of Spatial Durbin Model

In this paper, the inverse distance square weight matrix is used as the spatial weight matrix, the spatial Durbin model is selected, and stata15 is used for regression operation. The specific estimation results of relevant models are shown in Table 4.

From the regression model, we can see that the spatial Durbin model R-squared = 0.6567, compared with the simple panel data model-fitting, has improved, and each variable is significantly better. In the spatial Durbin model, independent variables not only have direct influence on the explained variables in the local area (i.e., direct effect), but also may have indirect influence on the explained variables in the neighboring area (i.e., indirect effect or spatial spillover effects). The total effect is the sum of direct and indirect effects. Therefore, the relationship between variables should be reflected through direct effect and indirect effect.

It can be seen from the above table that in the direct effect of the Spatial Durbin model, compared with the panel data model, the impact of PM2.5 annual average concentration on the proportion of the health expenditure of local rural residents in consumption expenditure is no longer significant. The number of medical staff per 10,000 people and the total dependency ratio have a significant positive impact on the proportion of the health expenditure of rural residents in consumption expenditure, and the number of medical staff per 10,000 people has a greater impact, with an impact coefficient of 2.665. Like the panel regression model, the Engel coefficient of rural residents has a reverse effect on the proportion of the health expenditure of rural residents in consumption expenditure, and the influence coefficient is −0.120, which is less than that of the panel regression model. The number of beds per 10,000 people in medical institutions has a little inhibitory effect on the proportion of the health expenditure of rural residents in consumption expenditure. The direct effect of spatial Durbin model is generally smaller than that of panel data model, which indicates that panel data model overestimates the direct effect of the independent variables because it does not consider spatial spillover effect. The direct effect shows that AAC-PM2.5 has a statistically significant positive effect on the proportion of the health expenditure of rural residents in consumption expenditure, but the direct effect of PM2.5 pollution on the proportion of the health expenditure of rural residents in consumption expenditure is not significant. The reason may be that the sample selected in this paper is too short, or the average data processing method weakens the influence relationship between variables, etc.

In the indirect effect, the results show that AAC-PM2.5 and the number of health technicians per ten thousand population in the adjacent areas will have a positive impact on the proportion of health expenditure of local rural residents in consumption expenditure, and the influence of PM2.5 on the dependent variable is the most obvious, with the influence coefficient of 2.546. Air flow tends to radiate pollutants to the surrounding cities, thus affecting the health of residents in adjacent areas. The higher the availability of medical resources in adjacent areas is, the more convenient the surrounding residents are to receive medical and health services. Therefore, the number of health technicians per 10,000 people will affect the health expenditure of rural residents. The Engel coefficient of rural households still has inhibitory effect on the proportion of the health expenditure of rural residents in consumption expenditure, the coefficient is −0.054, which is significantly reduced compared with the direct effect. In the total effect, the average annual PM2.5 concentration, the number of health technicians per 10,000 population, and the total dependency ratio all have significant positive effects on the proportion of the health expenditure of rural residents in consumption expenditure. The reasons behind these effects may be that the negative impact of environmental pollution on health makes the potential health hazards appear. Moreover, the negative impact of environmental pollution on health is more obvious in the elderly, children, and other vulnerable groups. National health awareness is gradually enhanced, and more attention is paid to medical care. The rich medical care resources and market-oriented medical services have realized the medical demand of the whole people, which directly leads to increased medical care expenditure.

In a word, the regression results of the Spatial Durbin model show that the spatial spillover effect of PM2.5 pollution on the health expenditure of rural residents is greater than the direct effect after decomposing the impact effect of PM2.5 pollution on the health expenditure of rural residents into direct effect and spatial spillover effect. Therefore, the spatial spillover effect of PM2.5 pollution on the health expenditure of rural residents is particularly obvious. Specifically, under the intervention of Engel coefficient of rural families, dependency ratio of the old and young population and the number of health technicians per 10,000 population, AAC-PM2.5 has no significant impact on the growth of the proportion of the health expenditure of rural residents in consumption expenditure. In the total effect, AAC-PM2.5 also promoted the growth of the proportion of the health expenditure of rural residents in consumption expenditure, indicating that there is a causal relationship between air pollution and the growth of health expenditure. The higher the Engel coefficient is, the less the proportion of health expenditure is. The number of health technical personnel per 10,000 population has a significant role in promoting the growth of the proportion of health expenditure in consumption expenditure of rural residents in this region and adjacent areas. Increasing the training of professional health personnel is conducive to releasing the medical and health needs of rural residents.

## 4. Discussion and Conclusions

With the gradual enhancement of residents’ awareness of health and environmental protection, air pollution and national health problems have aroused more and more social concerns. Balancing the relationship between environmental pollution and healthcare expenditure has become a necessary issue in the process of human survival and development. Based on previous studies and based on provincial panel data from 2002 to 2015, this paper introduces spatial dimension and uses Spatial Durbin model to examine the spatial spillover effect of environmental pollution on health expenditure of rural residents. The main conclusions are as follows.

First of all, the spatial spillover effect between PM2.5 annual average concentration and rural residents’ health expenditure in neighboring areas is significantly positively correlated at a significant level of 5% (Coef: 2.546, Z: 2.340, see Table 4), that is, AAC-PM2.5 has a spatial spillover effect on the health expenditure of rural residents, which shows that the spatial spillover effect is greater than the direct effect, and the air flow tends to radiate PM2.5 pollutants to surrounding cities, thus affecting the health of residents in neighboring areas. This is consistent with the research conclusion of Xie (2016), Li et al. (2019) that the migration and diffusion of PM2.5 pollution pollutants will affect the air quality of surrounding provinces and cities, resulting in a region’s health risks not only from the local PM2.5 pollution, but also a large part of the risk from the nearby PM2.5 pollution.

Secondly, the coefficient of influence of industrial wastewater and waste gas emissions per unit area on the proportion of health expenditure to consumption expenditure of rural residents is 0, which is contrary to the view that industrial waste water and waste gas emissions affect health expenditure in existing studies. The reason for this result may be that the average industrial wastewater and waste gas emissions per unit area do not reach the threshold value that causes significant changes in health expenditure, as the research of scholar He (2011) [33] found: significant changes in medical expenditure can only be caused by a large number of environmental changes, that is, environmental deterioration or improvement to a certain extent will significantly affect medical expenditure. The threshold for the impact of environmental pollution on health care spending will be the subject of subsequent studies.

Therefore, the above conclusions provide us with some policy implications. In view of the current situation of the significant growth of medical and health expenditure in recent years, we can intervene from three aspects: natural environment, medical and health resources, and individual residents, so as to slow down the upward pressure of medical and health expenditure and urge the healthy development of medical and health expenditure. Firstly, on the natural environment pollution for the negative impact of residents health care expenditure, the department concerned should fully implement the atmospheric pollution control and prevention, treatment, and resolutely implement the “polluter governance”, strengthen ecological environmental protection, good pollution control to be completed, to reduce the negative impact of environmental pollution on residents health, reduce personal and medical insurance fund has public health care spending. Secondly, the government needs to strengthen the training of professional health technicians, vigorously cultivate top-notch talents in the medical and health field, rationally distribute medical and health resources, and provide basic guarantees for releasing the medical and health needs of rural residents. Finally, the government needs to pay more attention to rural residents, the vulnerable groups, and their opportunities to share the fruits of economic development equally. Governments also need to help migrant workers find jobs and ensure their income, in particular by providing critical illness relief and other support, and by ending poverty caused by illness and consolidating the success of comprehensive poverty reduction.

## Figures and Tables

**Table 1:** Descriptive statistics of variables.

Variable	Variable Meaning	Obs	Mean	Std. Dev.	Min	Max
Y	The proportion of the health expenditure of rural residents in consumption expenditure	434	7.418	2.301	1.9	14
lnx	Logarithm of annual mean concentration of PM2.5	434	3.5	0.583	1.412	4.426
Water	Industrial wastewater discharge per unit area	434	7837.375	14,065.871	2.875	102,947.62
Gas	Industrial waste gas emission per unit area	434	1639.32	3191.183	0.106	21,752.857
Ec	Engel coefficient of rural household	434	41.713	7.315	27.5	65.1
Doctor	Number of health technicians per 10,000 population	434	0.465	0.188	0.2	1.546
Sickbed	Number of beds per 10,000 people in medical institutions	434	38.201	25.188	2.188	302.964
Dr	Sum of dependency ratio of young and old	434	37.048	6.969	19.27	57.58

**Table 2:** Regression results of panel data.

Y	Coef.	SE (Standard Error)	*p*-Value
lnx	0.862	0.363	0.018 **
Ec	−0.157	0.014	0.000 ***
Water	0.000	0.000	0.015 **
Gas	0.000	0.000	0.000 ***
Doctor	4.045	0.761	0.000 ***
Sickbed	−0.004	0.002	0.124
Dr	−0.005	0.017	0.747
Constant	8.180	1.870	0.000 ***
R-squared	0.641	Prob > F	0.000 ***

Note: ***, ** represent significant at 1%, 5%, respectively.

**Table 3:** Moran’s I test results of the proportion of the health expenditure in consumption expenditure of rural residents and the annual average concentration of PM2.5.

Year	Y		lnx	
	M	S	M	S
2002	0.257	0	0.176	0.001
2003	0.339	0	0.203	0
2004	0.233	0	0.236	0
2005	0.138	0.008	0.219	0
2006	0.08	0.059	0.251	0
2007	0.186	0.001	0.195	0
2008	0.214	0	0.203	0
2009	0.223	0	0.218	0
2010	0.256	0	0.213	0
2011	0.171	0.002	0.184	0.001
2012	0.2	0	0.203	0
2013	0.101	0.03	0.194	0.001
2014	0.151	0.005	0.215	0
2015	0.118	0.015	0.233	0

Notes: M = Moran’s I; S = Significance.

**Table 4:** Regression results of spatial Durbin model.

**Direct Effect**	**Coef.**	**SE (Standard Error)**	**Z**	**P > z**
lnx	0.070	0.431	0.160	0.872
Ec	−0.120	0.014	−8.540	0.000
Water	0.000	0.000	2.440	0.015
Gas	0.000	0.000	5.580	0.000
Doctor	2.665	0.754	3.530	0.000
Sickbed	−0.005	0.002	−2.160	0.031
Dr	0.031	0.018	1.760	0.079
**Indirect Effect** **(Spatial Spillover Effects)**	**Coef.**	**SE (Standard Error)**	**Z**	**P > z**
lnx	2.546	1.086	2.340	0.019
Ec	−0.054	0.014	−3.910	0.000
Water	0.000	0.000	2.080	0.038
Gas	0.000	0.000	3.270	0.001
Doctor	1.170	0.392	2.990	0.003
Sickbed	−0.002	0.001	−1.760	0.079
Dr	0.015	0.010	1.430	0.153
**Total Effect**	**Coef.**	**SE (Standard Error)**	**Z**	**P > z**
lnx	2.616	0.959	2.730	0.006
Ec	−0.174	0.020	−8.790	0.000
Water	0.000	0.000	2.420	0.015
Gas	0.001	0.000	5.370	0.000
Doctor	3.835	1.039	3.690	0.000
Sickbed	−0.007	0.003	−2.090	0.037
Dr	0.046	0.027	1.680	0.094
R-squared	0.6567			

## Data Availability

All data generated or analyzed during this study are included in this published article.
